# Diagnostic value of a computer-assisted diagnosis system for the ultrasound features in thyroid nodules

**DOI:** 10.20945/2359-4292-2022-0501

**Published:** 2023-11-10

**Authors:** Yiwei Wang, Ming Yu, Minliang He, Ganjun Zhang, Libo Zhang, Bo Zhang

**Affiliations:** 1 Graduate School of Dalian Medical University Dalian Liaoning China Graduate School of Dalian Medical University, Dalian, Liaoning, China; 2 Tend. AI Medical Technology China Tend. AI Medical Technology, China; 3 Shanghai East Hospital Department of Ultrasound in Medicine Shanghai China Shanghai East Hospital, Department of Ultrasound in Medicine, Shanghai, China; 4 Tongji University School of Medicine Shanghai East Hospital Department of Ultrasound in Medicine Shanghai China Shanghai East Hospital, Tongji University School of Medicine, Department of Ultrasound in Medicine, Shanghai, China

**Keywords:** Thyroid nodules, ultrasound, computer-aided diagnosis system, diagnosis

## Abstract

**Objective:**

To explore the diagnostic value of the TUIAS (SW_TH01/II) computer-aided diagnosis (CAD) software system for the ultrasound Thyroid Imaging Reporting and Data System (TI-RADS) features in thyroid nodules.

**Materials and methods:**

This retrospective study enrolled patients with thyroid nodules in Shanghai East Hospital between January 2017 and October 2021. The novel CAD software (SW_TH01/II) and three sonographers performed a qualitative analysis of the ultrasound TI-RADS features in aspect ratio, margin irregularity, margin smoothness, calcification, and echogenicity of the thyroid nodules.

**Results:**

A total of 225 patients were enrolled. The accuracy, sensitivity, and specificity of the CAD software in “aspect ratio” were 95.6%, 96.2%, and 95.4%, in “margin irregularity” were 90.7%, 90.5%, and 90.9%, in “margin smoothness” were 85.8%, 88.5%, and 83.0%, in “calcification” were 83.6%, 81.7%, and 82.0%, in “homogeneity” were 88.9%, 90.6%, and 82.2%, in “major echo” were 85.3%, 88.0%, and 85.4%, and in “contains very hypoechoic echo” were 92.0%, 90.0%, and 92.4%. The analysis time of the CAD software was significantly shorter than for the sonographers (2.7 ± 1.6 vs. 29.7 ± 12.7 s, P < 0.001).

**Conclusion:**

The CAD system achieved high accuracy in describing thyroid nodule features. It might assist in clinical thyroid nodule analysis.

## INTRODUCTION

A thyroid nodule is a discrete lesion in the thyroid gland that is radiologically distinct from surrounding normal thyroid tissue (1,2). Thyroid nodules are detected in up to 50%-65% of healthy individuals (3). They are four times more common in women than in men and occur more frequently with increasing age (2,4). Most thyroid nodules are asymptomatic; palpable nodules often are discovered on physical exam, and nonpalpable nodules frequently are detected incidentally on imaging studies performed for unrelated reasons (2,4). Symptomatic patients can complain of symptoms related to hyperthyroidism or hypothyroidism (in about 5% of cases), compressive symptoms (in about 5% of cases), or cosmetic concerns. Thyroid nodules may be caused by both benign (about 90%) and malignant (about 10%) lesions (2,4). Risk factors for malignancy include a family history of thyroid cancer and a history of radiation (1,2,4,5). While thyroid nodules can be associated with thyroid dysfunction or local mass effects, the primary clinical concern is to identify and treat lesions that are malignant or at high risk for malignancy (1,4).

Ultrasound (US) imaging is the first choice for diagnosing thyroid nodules as it visualizes soft tissue structures without harmful radiation (1,2,4). US is the most widely available and affordable imaging modality (6), but its diagnostic accuracy can be highly related to physician experience and is operator-dependent (7). Computer-aided diagnosis (CAD) systems could be a way to mitigate subjectivity and operator-dependency. Most of the CAD systems being developed currently focus on classifying benign and malignant nodules only. Such a binary classification is not informative enough to fulfill the clinical requirements. Most of the state-of-art artificial intelligence (AI) models (*e.g.*, deep neural networks) developed in a research endeavor contain a huge number of model parameters; the meaning and impact of each of these parameters is difficult to interpret, and such models are hence known as “black-box” models. Therefore, most CAD systems are widely deployed in real-life clinics due to the lack of evidence on how diagnostic decisions are made (8). In bridging the gap between theory and practice, TenD.AI Medical Technology has recently developed an innovative CAD system (TUIAS, SW_TH01/II) to assist US thyroid nodule assessment. The software predicts different descriptors from the thyroid nodule with a scheme developed by the ACR Thyroid Imaging Reporting and Data System (TI-RADS) (9) and some other similar guidelines (10,11). Compared to conventional methods, the software could assist decision-making.

This study aimed to explore the diagnostic values of the TUIAS (SW_TH01/II) CAD software system for the ultrasound TI-RADS features in thyroid nodule. The results could provide evidence for its use in clinics.

## MATERIALS AND METHODS

### Study design and participants

This retrospective study enrolled patients with thyroid nodules in Shanghai East Hospital between January 2017 and October 2021. The inclusion criteria were 1) underwent two-dimensional US examination of the thyroid with transverse and longitudinal views, and 2) with thyroid nodule detected by US. The exclusion criteria were 1) incomplete US images of the thyroid gland or 2) Hashimoto's thyroiditis, subacute thyroiditis, Graves’ disease, or other diseases with ununiform background echoes. This study was approved by the Medical Research Ethics Committee of Shanghai East Hospital (No. 2021-038). The requirement for informed consent was waived as this was a retrospective study.

### Data collection

**Figure 1:**
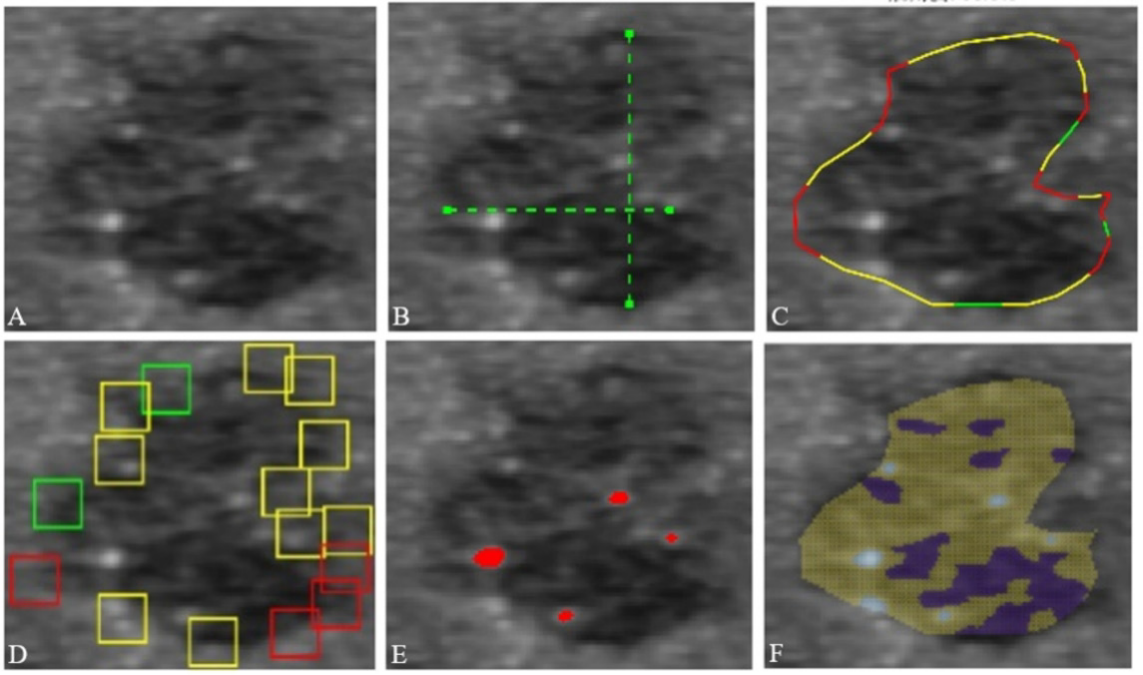
TI-RADS-based descriptors output by the CAD software. (**A**) The original image of ROI in the thyroid. (**B**) Aspect ratio: taller than wide. (**C**) Margin irregularity: irregular. (**D**) Margin smoothness: unclear. (**E**) Calcification: micro calcification. (**F**) Echogenicity output. Homogeneity: no; major echo: hypoechoic; contains very hypoechoic echo: yes. TI-RADS: Thyroid Imaging Reporting and Data System; CAD: computer-aided diagnosis.

The US images of the thyroid nodules were collected from four ultrasound instruments (Philips IE33, Netherlands, linear probe L12-5, frequency 5-12 MHZ; Toshiba Aplio300 and 500, Japan, linear probe 14L5, frequency 5-14 MHz; Shenzhen, China, Mindrai, linear probe L14-5, frequency 9-14 MHz). The CAD software (TUIAS, SW_TH01/II) was used to delineate the region of interest (ROI) on the US images of thyroid nodules and perform a qualitative analysis to produce five main features of thyroid nodules according to the TI-RADS standard (9): aspect ratio, shape irregularity, margin smoothness, calcification, and echogenicity (Figure 1).

More specifically, the aspect ratio was classified as “taller than wide” or “wider than tall” by comparing the width and height of the nodule. The margin irregularity was classified as “regular” and “irregular”. Nodules were classified as “regular” if they were in a near oval or round shape without any lobulations or angulations; otherwise, they were classified as “irregular”. The margin smoothness was classified as “clear” or “not clear”, depending on the distinctiveness of the nodule margin. A “clear” margin was defined as sharp changes around the nodule boundary, whereas an “unclear” margin was defined as observing indistinguishable changes around the nodule boundary. The calcification descriptor indicated that the nodule had “no calcification”, “microcalcification”, or “macro calcification”, which was identified by the algorithm by studying the shape, size, brightness, and contrast of the local regions of suspected calcification. It should be noted that nodules containing both micro- and macrocalcifications were labeled as “microcalcification” for consistency. The echogenicity properties were described via three sub-descriptors respectively for “homogeneity”, “major echo”, and “containing very hypoechoic echo”. More specifically, the “homogeneity” was classified as either “yes” or “no”, depending on the type of echo that the nodule had. The “major echo” was determined by the type of echo that occupied the largest proportion of the nodule and was described as “anechoic”, “very hypoechoic”, “hypoechoic”, “isoechoic”, or “hyperechoic”. For the “containing very low echo” descriptor, the nodule containing very hypoechoic echo was described as “yes”; otherwise, “no”. The very low echo is an important indicator for the malignant tendency of thyroid nodules, which is therefore included as an echo feature. Currently, the five characteristics of thyroid nodules were determined and obtained by TI-RADS. In the future, the risk stratification can be synthesized in one click from TI-RADS.

Each feature was obtained by a specifically designed algorithm that took an ROI image as input and produced the appropriate label as the output. In addition, the algorithm also provided localization outputs, which were used by the software interface to visualize the locations of the descriptions. An example of an input ROI image and the feature outputs from the algorithms are shown in Figure 1. The computation time of the CAD software to generate the feature descriptors was recorded.

**Figure 2:**
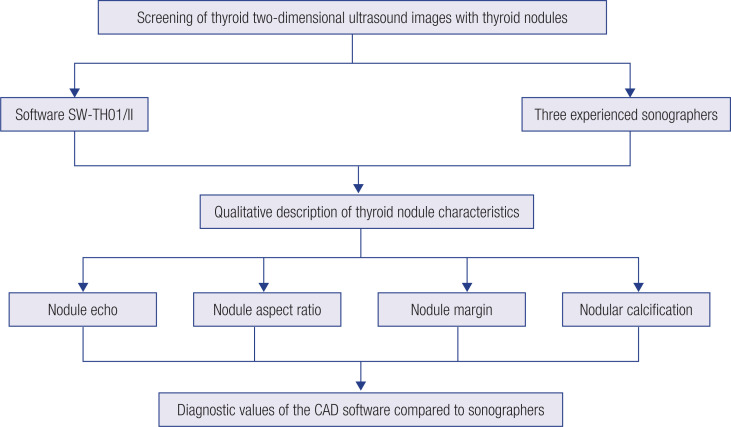
Schematic of the study design.

Three sonographers with vice senior and senior professional titles and at least 10 years of working experience in thyroid ultrasound diagnosis in Shanghai East Hospital participated in image delineation and produced the same TI-RADS-based feature descriptors of each nodule. The diagnosis reports issued by the three senior sonographers were used as the gold standard. Two sonographers first labeled the same US image independently. If both sonographers produced the same label, it was directly used as the final label; otherwise, the third sonographer participated in making the final decision on the final label for the image. The time for each sonographer to generate the imaging descriptors was also recorded. Afterward, the predicted results from the software were compared with the descriptors assigned by sonographers to evaluate diagnosis values and time efficiency (Figure 2).

### Statistical analysis

Statistical analyses were performed using SPSS 18 (SPSS, Armonk, NY, USA). Continuous variables with a normal distribution were described as means ± standard deviation (SD) and were compared with Student's t test. Categorical variables were described as n (%). Sensitivity, specificity, and accuracy were used to assess the diagnostic values of the software. Two-sided P-values <0.05 were considered statistically significant.

## RESULTS

This study enrolled 225 patients with thyroid nodules (Table 1). A total of 173 (76.9%) nodules were wider than tall, and 137 (60.9%) had irregular margins. One hundred and twelve (49.8%) nodules had clear margins. A total of 169 (75.1%) did not contain calcification and 45 (20.0%) nodules were homogeneous. For major echoes, 115 (51.1%) nodules were hypoechoic, and 54 (24.0%) nodules were isoechoic. A total of 185 (82.2%) did not contain hypoechoic echo.

**Table 1:** Ultrasound features of the thyroid nodules

TI-RADS features		n (%)
Aspect ratio		
	Taller than wide	52 (23.1%)
	Wider than tall	173 (76.9%)
Margin regularity		
	Regular	88 (39.1%)
	Irregular	137 (60.9%)
Margin smoothness		
	Clear	112 (49.8%)
	Unclear	113 (50.2%)
Calcification		
	Macrocalcification	6 (2.7%)
	Microcalcification	50 (22.2%)
	No calcification	169 (75.1%)
Echoic homogeneity		
	Yes	45 (20.0%)
	No	180 (80.0%)
Major echo		
	Anechoic	34 (15.1%)
	Very hypoechoic	19 (8.4%)
	Hypoechoic	115 (51.1%)
	Isoechoic	54 (24.0%)
	Hyperechoic	3 (1.3%)
Contains very hypoechoic echo		
	Yes	40 (17.8%)
	No	185 (82.2%)

TI-RADS: Thyroid Imaging Reporting and Data System

The accuracy, sensitivity, and specificity of the CAD software in “aspect ratio” were 95.6%, 96.2%, and 95.4%, in “margin irregularity” were 90.7%, 90.5%, and 90.9%, in “margin smoothness” were 85.8%, 88.5%, and 83.0%, in “calcification” were 83.6%, 81.7%, and 82.0%, in “homogeneity” were 88.9%, 90.6%, and 82.2%, in “major echo” were 85.3%, 88.0%, and 85.4%, and in “contains very hypoechoic echo” were 92.0%, 90.0%, and 92.4% (Table 2). The analysis time of the CAD software was significantly shorter than for the sonographers (2.7 ± 1.6 *vs.* 29.7 ± 12.7 s, P < 0.001).

**Table 2:** Diagnostic value of the CAD software in thyroid nodule features

	Accuracy	Sensitivity	Specificity
Aspect ratio	95.6%	96.2%	95.4%
Margin irregularity	90.7%	90.5%	90.9%
Margin smoothness	85.8%	88.5%	83.0%
Calcification	83.6%	81.7%	82.0%
Homogeneity	88.9%	90.6%	82.2%
Major echo	85.3%	88.0%	85.4%
Contains very hypoechoic echo	92.0%	90.0%	92.4%

## Discussion

This study showed that the accuracy, sensitivity, and specificity of the CAD software in describing thyroid nodule features were > 80%. The analysis time of the CAD software was significantly shorter than for the sonographers. The CAD system might assist in clinical thyroid nodule analysis, especially for junior doctors.

The American Thyroid Association (ATA) defines a thyroid nodule as a discrete lesion on the thyroid gland. With the help of imaging examinations, it can be observed that the nodule is different from the normal thyroid tissue structure and has relative boundaries. In recent years, the incidence of thyroid nodules has continued to increase. It is essential to determine the benign/malignant nature of the nodule. US has many advantages such as non-invasive, no radiation, low cost, and real-time imaging. It plays a crucial role in the detection and characterization of thyroid nodules. Still, assessments made by sonographers can be subjective, time-consuming, and usually influenced by human experience and the quality of the ultrasound equipment (3).

The first CAD systems were used to diagnose breast tumors in the 1960s, and AI-based CAD systems were developed to help sonographers analyze images, shorten the time cost of the diagnostic process, and reduce inter-observer variability (12). Along with the development of computer technology, image-based diagnostic techniques have been widely used in helping physicians identify thyroid nodules and other problems (13). Most of the current CAD systems mainly have two functions: (1) identifying benign *vs.* malignant thyroid nodules and (2) identifying nodule boundaries (14). At the moment, it remains controversial whether the diagnostic performance of conventional CAD systems can be comparable to experienced sonographers (15). There are many studies and applications for using neural networks for CAD (16). Four studies showed that US CAD systems improved the diagnostic accuracy of thyroid US and could help junior physicians make a diagnosis (17–20). Nevertheless, despite their impressive levels of accuracy, neural networks are essentially a “black box”, and they cannot give reasonable explanations for the reliability of predictions since they are based on a multiplicity of features not perceptible to the naked eye (21), hindering their wide use in clinical practice.

In this study, computer software was used to predict different nodule descriptors coherent with the TI-RADS. Within the past decades, most of the developed computer-vision-based thyroid US image classification algorithms produce a single binary output only, as either benign or malignant (22). However, the real practical requirement in medicine is the ability to interpret algorithmic decisions and improve the confidence of diagnostic results (23). As a potential solution, the results produced by the tested software can offer great interpretability to the clinical decision made. In addition, the world is currently known for its unequal distribution of medical resources, where large gaps exist across different geographical regions (24), and disparities are also seen within large countries like China (25). Some sonographers from poor regions may have limited technical resources and are short on experience, which can easily lead to misdiagnosis of thyroid nodules. The tested software can potentially benefit thyroid nodule screening programs, especially in the region facing poverty and healthcare access issues (23).

Apart from the potential benefits of using the tested software, it is also important to understand that there are still limitations. First, the software can only analyze a single image for each nodule. Human intervention is still required when selecting images (26), and the ROI selected can be affected by the experience and skill of the operating physician. At present, only static images of thyroid nodules can be analyzed instead of real-time video footage. In addition to that, the tested software cannot analyze blood flow signals of thyroid nodules and the features of cervical lymph nodes.

Nowadays, with the rapid development of CAD technology, there is still a huge potential for improving the US CAD systems for analyzing thyroid nodules. In the future, CAD software can still improve by combining current features with analysis results on cervical lymph node characteristics and blood flow signals. Developing a CAD system that analyses US footage in real-time is urgently required and can be one of the very important research directions in the future. It can also be combined with other techniques such as contrast-enhanced ultrasound (CEUS) and ultrasound elastography. CEUS helps the physicians understand the blood perfusion and hemodynamic changes in the ROI by showing the movement and distribution of the contrast agent microbubbles. The solidity difference in tissue is important for evaluating the benignity or malignancy of the tissue. It will help the physician derive more accurate and speedy diagnoses if these new ultrasound technologies can be fully integrated within current state-of-art CAD systems.

There were several limitations in the present study. This study was a single-centered retrospective study with a small sample size, which requires further confirmation in larger studies. The TI-RADS classification of the thyroid nodules enrolled were not collected. Further studies are needed to explore the diagnostic value of the CAD systems for TI-RADS classification.

This study preliminarily explored the accuracy, sensitivity, and specificity of using CAD software (TUIAS, SW_TH01/II) for analyzing the TI-RADS features of thyroid nodules based on static images. Among all descriptors derived, margins irregularity and aspect ratios had the highest diagnostic accuracy, more than 90%, and the accuracy was >80% for all features. The time efficacy of analyzing thyroid nodules using the software was also significantly better than for physicians. Therefore, using the software might assist sonographers in analyzing thyroid nodules with higher efficiency and consistency.
